# Autocrine PDGF stimulation in malignancies

**DOI:** 10.3109/03009734.2012.658119

**Published:** 2012-04-19

**Authors:** Carl-Henrik Heldin

**Affiliations:** Ludwig Institute for Cancer Research, Uppsala University, BMC, Box 595, S-751 24 Uppsala, Sweden

**Keywords:** Molecular biology, oncogenes, tumor biology

## Abstract

Platelet-derived growth factor (PDGF) isoforms are important mitogens for different types of mesenchymal cells, which have important functions during the embryonal development and in the adult during wound healing and tissue homeostasis. In tumors, PDGF isoforms are often over-expressed and contribute to the growth of both normal and malignant cells. This review focuses on tumors expressing PDGF isoforms together with their tyrosine kinase receptors, thus resulting in autocrine stimulation of growth and survival. Patients with such tumors could benefit from treatment with inhibitors of either PDGF or PDGF receptors.

## Introduction

Members of the platelet-derived growth factor (PDGF) family are major mitogens for connective tissue cells, glial cells, and certain other cell types. Structurally they are homodimers of related A-, B-, C-, and D-polypeptide chains, and an AB heterodimer (1).

PDGF isoforms exert their cellular effects by binding to α- and β-tyrosine kinase receptors. Ligand binding induces dimerization of the receptors; the α-receptor binds all PDGF chains except the D-chain, whereas the β-receptor binds the B- and D-chains. Thus, the different PDGF isoforms induce different homo- and heterodimeric complexes of α- and β-receptors. In the dimeric receptor complexes, receptors phosphorylate each other in trans positions on specific tyrosine residues, which allows binding and activation of SH2-domain-containing signaling molecules (1). Some of these have enzymatic activities, such as phospholipase Cγ (PLCγ), the tyrosine kinase Src, the protein tyrosine phosphatase SHP-2, and the GTPase activating protein for Ras (RasGAP). Alternatively, they are adaptor molecules forming complexes with enzymes, such as Grb2, which forms a complex with SOS1, a nucleotide exchange molecule that activates Ras, and the p85 regulatory subunit of phosphatidylinositol 3'-kinase (PI3-kinase), which forms a complex with the p110 catalytic subunit. In addition, members of the STAT family of transcription factors bind to activated PDGF receptors, as do the adaptor molecules Grb7, Shc, Nck, and Crk.

PDGF isoforms stimulate proliferation, survival, chemotaxis, and differentiation of cells. They have important functions during embryonic development (2), and in the adult during wound healing (3) and in the control of interstitial fluid pressure (4). Over-activity of PDGF has been linked to several pathological conditions, including malignancies and other conditions involving an excess cell proliferation, such as fibrotic conditions and atherosclerosis (1).

PDGF isoforms are often over-expressed in malignancies and contribute to the growth of certain tumor types as well as to non-transformed cells in solid tumors, such as pericytes and smooth muscle cells of vessels and of stromal fibroblasts. Certain PDGF receptor-bearing tumor cells produce PDGF isoforms, which stimulate cell growth and survival in an autocrine manner. The aim of the present communication is to review the involvement of autocrine PDGF stimulation in malignancies.

## Homology between PDGF and the Sis oncogene product

PDGF was originally purified from human platelets (5–8). When the purified PDGF was subjected to amino acid sequencing, a homology to the product of the oncogene *sis* was noticed (9,10). In fact, the gene for the B-chain of PDGF has been transduced by the simian sarcoma virus (SSV), and infected cells were shown to produce large amounts of a PDGF-BB-like growth factor (11,12). Evidence that the autocrine stimulation is crucial for cell transformation was rapidly obtained, e.g. it was shown that the transformed phenotype of SSV-transformed fibroblasts can be normalized by inhibitory PDGF antibodies (13).

The discovery of the homology between PDGF and Sis was rapidly followed by additional findings of homologies between products of retroviral oncogenes and growth factor receptors, as well as with components of their intracellular pathways. Together, these observations provided strong support for the hypothesis that oncogenes transform cells by subverting the mitogenic pathways of growth factors (14). Furthermore, the findings triggered intensive efforts to investigate if autocrine mechanisms occur also in human malignancies.

## Autocrine PDGF stimulation in human glioma, osteosarcoma, and other tumor types

During the 1970s, a hypothesis was formulated that tumor cells may make their own growth factors and thereby be self-sufficient with regard to growth stimulatory signals (15). To explore this hypothesis, a growth factor produced by the human osteosarcoma cell line U-2OS was purified (16,17). Initial characterization revealed that this factor was similar but not identical to PDGF purified from platelets; sequencing showed that it was in fact PDGF-AA, whereas platelets contain mainly PDGF-AB (18). Autocrine PDGF receptor activation was demonstrated in U-2OS cells, but effects on growth stimulation were more difficult to show, probably because of the numerous other mutations these cells have acquired during many years of *in-vitro* culturing (19). Similar analyses of glioma cell lines revealed that co-expression of PDGF isoforms and PDGF receptors is common, suggesting autocrine mechanisms (20–24). Furthermore, analysis of expression of PDGF isoforms and PDGF receptors in sections of human glioblastomas provided evidence that both types of PDGF receptors are involved in autocrine and paracrine growth stimulation of gliomas, affecting different cellular compartments, however. Thus, the α-receptor is expressed mainly in the tumor cells, whereas the β-receptor is expressed in cells of the supporting stroma (25–29). The levels of expression of PDGF ligands as well as receptors are higher in more malignant tumors, suggesting that autocrine and paracrine effects of PDGF increase with degree of malignancy. Gliomas are probably the tumor type in which PDGF autocrine mechanisms are most important, and nearly 30% of human gliomas show over-activity of PDGF receptor signaling (30). Gliomas are discussed further by Lindberg and Holland (31) in this series.

PDGF has also been implicated in autocrine mechanisms of other tumor types. Thus, malignancy-dependent expressions of PDGF and PDGF receptors were observed in sarcomas (32,33). Co-expression of PDGF and PDGF receptors has also been reported in an AIDS-related Kaposi's sarcoma (34) and in meningeomas (35,36). Moreover, an autocrine PDGF-BB/PDGF β-receptor loop was found to mediate survival of large granular lymphocyte leukemia of both T- and NK-cell origin (37). In addition, co-expression of PDGF-AA and PDGF α-receptor in the epithelial part of Wilms' tumor of the kidney is common; in contrast to other tumors with autocrine PDGF stimulation, the expression of PDGF-A and PDGF α-receptor in Wilms' tumor correlates to favorable prognosis (38).

Screening of 637 human tumor-derived cell lines revealed that only 2 were sensitive to sunitinib, an inhibitor which targets the PDGF receptor kinases as well as other kinases, i.e. a non-small-cell lung cancer and a rhabdomyosarcoma (39). Both these cell lines co-express the PDGF α-receptor and PDGF-C. Moreover, investigation of a large number of human and mouse rhabdomyosarcomas revealed that the PDGF α-receptor is a target of the Pax3/Fkhr chimeric transcription factor, which is found in a majority of this tumor type (40). This results in over-expression of the PDGF α-receptor, which is correlated to poor prognosis (41), and often occurs together with expression of PDGF-A or -C, thus creating autocrine loops.

In the rare skin tumor dermatofibrosarcoma protuberans (DFSP), a specific genetic perturbation is responsible for the establishment of autocrine PDGF stimulation. Thus, in this disease the PDGF-B gene is fused to the collagen 1A1 gene, leading to the production of a collagen 1A1/PDGF-B fusion protein, which is processed to mature PDGF-BB that activates PDGF receptors on fibroblasts in an autocrine manner (42–45).

Taken together, there are thus now several examples of autocrine mechanisms involving PDGF and PDGF receptors in different forms of malignancies.

## Intracrine versus extracrine PDGF stimulation

**Figure 1.**
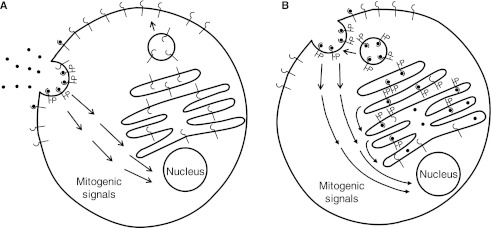
A: A PDGF receptor-bearing cell that does not produce PDGF itself responds to PDGF via activation of receptors at the cell surface. This initiates activation of intracellular signaling pathways leading to cell growth, proliferation, and survival. B: In a PDGF receptor-bearing cell that produces PDGF-A or -B, the ligands will meet and activate the receptors already in the ER, Golgi, and secretary vesicles. Whereas some intracellular signaling pathways are activated intracellularly, other pathways important for mitogenesis are not activated until the ligand–receptor complex reaches the cell membrane.

While all PDGF isoforms are produced as inactive precursor molecules, the N-terminal parts of PDGF-A and -B are removed already intracellularly by furin-like proteases. In contrast, PDGF-C and -D are secreted as latent precursor molecules containing N-terminal CUB domains, which need to be cleaved off by proteases before these PDGF isoforms can bind to receptors. Thus, tissue plasminogen activator (tPA) has been shown to cleave and activate PDGF-CC (46) and urokinase plasminogen activator (uPA) PDGF-DD (47), but other proteases may also be involved. Thus, in cells which express PDGF-AA, -AB, or -BB together with PDGF receptors, the active ligands will be present together with the extracellular, ligand-binding parts of the receptors in the endoplasmic reticulum, Golgi apparatus, and secretory vesicles (Figure 1). In such cells, there is evidence that receptors are activated intracellularly before they have obtained their mature glycosylation and have reached the cell surface (48-50). However, several observations support the notion that only a subset of the intracellular pathways can be activated in intracellular vesicles, and that the ligand–receptor complex needs to reach the cell surface before an efficient mitogenic signal is initiated (Figure 1) (13,51–53). One mechanistic explanation could be that certain signal transduction components, critical for the mitogenic response, are located at the plasma membrane. In support of this possibility, activated PDGF β-receptors in *sis*-transformed cells were found to interact with certain signaling molecules intracellularly, e.g. PLCγ, RasGAP, and PI3-kinase, whereas efficient interaction with SHP-2, Grb2, and Src occurred only after the receptor had reached the plasma membrane (54).

Interestingly, the C-terminals of PDGF-B and a long splice variant of PDGF-A contain basic amino acid sequences, which mediate binding to extracellular matrix molecules (55,56). Moreover, proteolytic activation of PDGF-DD was found to reveal a retention motif mediating interactions with pericellular components (57). The presence of these retention motifs restricts the action of the PDGF isoforms and thus enhances autocrine and paracrine stimulation of cells in the local environment, at the expense of stimulation of cells at a distance.

## Mutations of genes for PDGF receptors in human malignancies

In addition to classical autocrine stimulation, there are examples of mutations in the genes for PDGF receptors, which cause their activation and promote tumorigenesis. Thus, in chronic myelomonocytic leukemia (CMML), the kinase domain of the PDGF β-receptor is fused to different partners, e.g. the transcription factor Tel or rabaptin 5, which have in common that they can dimerize or oligomerize (58,59). Analogously, in patients with idiopathic hypereosinophilia, the kinase domain of the PDGF α-receptor is fused to FIP1L1 (60,61). A similar FIP1L1-PDGF α-receptor fusion has been observed in systemic mastocytosis (62). In addition to the juxtaposition of the kinase domains of the receptors, the loss of regulatory sequences in the juxtamembrane (63) and transmembrane (64) domains is important for the increased autophosphorylation and initiation of signaling pathways promoting cell growth and survival.

A majority of gastrointestinal stromal tumors (GISTs) have activating point mutations in Kit, a tyrosine kinase receptor for stem cell factor, which is structurally similar to PDGF receptors. However, some of these tumors are instead driven by point mutations in the PDGF α-receptor gene affecting the control mechanisms of the kinase; such mutations make the receptor kinase constitutively active (65).

Finally, the PDGF α-receptor has been found to be amplified in a subset of glioblastoma tumors (66–68), in anaplastic oligodendrogliomas (69), in esophageal squamous cell carcinoma (70), and in pulmonary artery intimal sarcoma (71). The large amounts of receptors expressed on such cells may cause constitutive activation of the receptors, since the high receptor density may promote ligand-independent receptor–receptor interactions. Alternatively, the cells at least become very sensitive to PDGF stimulation. In addition, a transforming deletion mutant of the PDGF α-receptor has been described in gliomas (72).

## Treatment of PDGF-dependent tumors with PDGF antagonists

Several types of PDGF antagonists have been developed, including antibodies and aptamers against PDGF or PDGF receptors, and low-molecular-weight inhibitors of the receptor kinases as reviewed by Östman and Heldin (73). A few kinase inhibitors have been approved for clinical use, including imatinib, which is fairly selective for PDGF receptors, Kit and Abl tyrosine kinases, and sorafinib and sunitinib which have broader specificities and inhibit also other kinases.

In the rather rare tumor types in which mutations of PDGF or PDGF receptor genes drive tumorigenesis, e.g. DFSP (74–76), CMML (77,78), hypereosinophilic syndrome (79), and GIST (80), treatment with imatinib has been shown to have beneficial effects. Whereas inhibition of glioma cell growth by imatinib or other PDGF receptor kinase inhibitors has been observed in animal tumor models (28,81,82), no clear benefit has been noticed when glioma patients have been treated with PDGF receptor kinase inhibitors, suggesting that in human glioma perturbations of PDGF signaling pathways are not of unique importance. Other genetic alterations have, however, occurred, which also drive tumor cell growth and survival.

In addition to a direct effect on tumor cells with over-active PDGF signaling, PDGF antagonists have also been shown be useful to target cells of the stroma of solid tumors (83).

## Multistep induction of malignancies

It is well established that the development of a fully malignant tumor requires several genetic or epigenetic alterations. It is therefore likely that autocrine PDGF stimulation is an initial event in tumor progression, which leads to an expansion of cells that are targets for neoplastic transformation. Alternatively, the aberrant production of PDGF may stimulate the growth of cells that are already genetically altered. In the case of SSV-induced transformation, there is also the possibility that the *sis* oncogene is inserted in regions of the genome where it affects the expression of oncogenes or tumor suppressor genes.

The possible importance of insertional mutagenesis in PDGF-driven gliomagenesis has been explored in Bengt Westermark's laboratory using a recombinant Moloney leukemia virus encoding the PDGF B-chain (84). Sixty-six common retroviral insertion sites were identified (85), and retroviral insertion was found to affect the expression of a number of genes with a potential role in the regulation of glial cell growth and survival (86). One of the targeted genes was the gene for cGMP-dependent protein kinase II; an anti-proliferative role of this kinase was demonstrated, which was lost during the loss-of-function retroviral insertion (87). Another common integration site was a gain-of-function insertion in the gene for the transcription factor Sox10 (88); over-expression of Sox10 was shown to enhance the tumorigenic activity of PDGF-B but did not alone induce gliomas. Integrations in the gene for Sox5 were also observed, but in this case suppression of Sox5 activity was correlated to gliomagenesis (89). Loss-of-function retroviral insertion in the gene for p190RhoGAP was also shown to promote glioma development, most likely via loss of control of Rho signaling (90).

In high-grade oligodendrogliomas, perturbation of PDGF, or epidermal growth factor, signaling is often accompanied by homozygous deletion of the *INK4a-ARF* locus (91,92). This locus encodes the tumor suppressor proteins p16^INK4a^ and p14^ARF^, which control the Rb and p53 pathways, respectively. In PDGF-induced oligodendroglioma development, loss of *Ink4a* was found to render astrocytes susceptible to PDGF-BB-induced tumorigenesis, whereas loss of *Arf* caused increased malignancy (93). Taken together, these observations illustrate that over-activity of PDGF receptors need to be complemented by other cellular alterations to promote tumorigenesis.

## Autocrine PDGF stimulation during epithelial-mesenchymal transition

Epithelial cells normally do not contain PDGF receptors. However, epithelial tumors can undergo epithelial-mesenchymal transition (EMT), a change in phenotype which makes the tumor cells more invasive and prone to make metastases (94). *In vitro*, EMT is promoted e.g. by stimulation by transforming growth factor-β (TGFβ), certain tyrosine kinase receptor ligands, and Notch. In conjunction with EMT, PDGF and PDGF receptors are induced (95). Interestingly, the metastatic potential of mammary epithelial tumors was shown to be dependent on an autocrine PDGF/PDGF receptor loop; inhibition of PDGF by a dominant negative receptor or by imatinib inhibited metastasis in mouse models (96). The invasiveness of human mammary carcinomas correlates to the expression of PDGF α- and β-receptors (97), and earlier studies had shown that the expression of PDGF correlates with unfavorable prognosis (98). Expression of PDGF and PDGF receptors also correlates to poor prognosis of lung carcinoma (99). Moreover, TGFβ-induced EMT of mouse hepatocellular carcinoma was found to involve expression of PDGF-AA and PDGF α-receptor and the establishment of an autocrine loop (99). In this tumor type, hypoxia was shown to induce PDGF-BB production via induction of HIF-1α (100).

PDGF-D and the PDGF β-receptor have been implicated in autocrine mechanisms in prostate cancer cell lines (101). Prostate cancer cells secrete matriptase, which activates PDGF-D by proteolytic removal of the CUB domain, thus inducing an autocrine stimulation. Moreover, immunohistochemical stainings of sections of human prostate cancers revealed co-staining of PDGF-D and matriptase (101). In PC-3 prostate carcinoma cells, PDGF-DD has furthermore been shown to drive the EMT process by repressing miR-200 which targets ZEB1, ZEB2, and Snail2, critical components of the EMT transcriptional program (102). A role for PDGF α-receptors in promoting metastasis of prostate cancer cells to bone has also been reported (103), and targeting the PDGF α-receptor with a monoclonal antibody dramatically inhibited the growth of skeletal prostate cancer metastases in an animal model (104). Evidence has been presented that in the latter case the PDGF α-receptor is not activated by ligand binding in a conventional way, but rather transactivated by an intracellular mechanism (105).

## Does autocrine PDGF stimulation occur in normal cells?

PDGF isoforms have important functions during embryonic development. Often the ligand is produced by epithelial or endothelial cells and acts on nearby mesenchymal cells in a paracrine manner (2). There are examples of non-transformed cell types which both can express PDGF receptors and produce PDGF, e.g. smooth muscle cells, endothelial cells, and macrophages (1,2). However, it is not clear whether normal cells express PDGF receptors and synthesize PDGF at the same time. If there are such examples of autocrine PDGF loops also in normal cells it is likely that they are transient and well controlled.

## Future perspectives

The discovery that the Sis oncogene product is similar to PDGF-B led to the first demonstration of an oncogenic autocrine mechanism. Subsequent studies have shown that autocrine PDGF loops occur in human tumors, both in e.g. gliomas and sarcomas where the corresponding normal cell type expresses PDGF receptors, in epithelial cells that have undergone EMT, and in some rare tumors which aberrantly express the PDGF receptors. Examples of autocrine mechanisms involving other growth factors and cytokines are also accumulating. Thus, it is likely that autocrine stimulation is common in tumors.

In addition to autocrine stimulation, PDGF is involved in paracrine stimulation of normal cells in solid tumors; PDGF made by tumor cells or other cells can thus act on pericytes, smooth muscle cells, and endothelial cells, thereby promoting angiogenesis, as well as on stromal fibroblasts and myofibroblasts, thereby controlling the interstitial fluid pressure of tumors (83). Paracrine mechanisms involving a number of different growth factors and cytokines with trophic effects on tumor cells as well as non-tumor cells have important roles in the balanced growth of tumor tissue and in the recruitment of other cell types to the tumor, including macrophages. Particularly M2 macrophages are well known to secrete many different growth factors and cytokines, thus creating a vicious cycle. The availability of sensitive and affordable microarray and proteomic techniques will make it possible in the future to perform systematic analysis of autocrine and paracrine mechanisms in human tumors. Such information will be important for optimal design of treatment.

Recent work supports the notion that tumor development is driven by a subpopulation of cells with self-regenerating capacity, so-called cancer stem cells. Importantly, PDGF-BB has been shown to promote expansion of neural stem/progenitor cells (106) and to sustain self-renewal and tumorigenicity of glioma cancer-initiating cells by preventing oligodendrocyte differentiation (107). Additional insights into the effect of PDGF on cancer stem cells are highly warranted.

The extensive autocrine and paracrine stimulations that occur in tumors, which are of crucial importance for the growth and survival of tumor cells, offer opportunities for selective treatment of tumor patients by targeting growth factors and their receptors. A few selective signal transduction antagonists have been approved for clinical use, and many others are under testing in clinical trials. It seems likely that such inhibitors will be useful tools in future treatment of tumor patients.
